# MYB Activates the Hedgehog Signaling Pathway to Repress Natural Killer Cytotoxicity in Cervical Cancer

**DOI:** 10.1002/kjm2.70084

**Published:** 2025-09-01

**Authors:** Yu Wang, Chen Li

**Affiliations:** ^1^ Molecular Testing Center The First Affiliated Hospital of Jinzhou Medical University Jinzhou China

**Keywords:** cervical cancer, hedgehog signaling pathway, MYB, natural killer cells

## Abstract

Natural killer (NK) cells present in the tumor microenvironment serve as a critical line of defense against various malignancies, including cervical cancer. While MYB is known to drive malignancy progression, its influence on NK cell activity remains poorly understood. This study aimed to elucidate the role of MYB in regulating NK cell cytotoxicity and its underlying mechanism in cervical cancer cells. MYB expression in cervical cancer tissues and cells was analyzed using bioinformatics and qRT‐PCR. Cell viability was assessed via CCK‐8 assay, while NK cell‐mediated killing of cervical cancer cells was evaluated through cytotoxicity assays. The expression levels of cytotoxic factors (IFN‐*γ* and TNF‐*α*) were measured by ELISA, whereas perforin and granzyme B were detected via immunofluorescence. Apoptosis was analyzed using flow cytometry. To investigate the impact of MYB on the hedgehog signaling pathway, the expression levels of related factors (PTCH1, Gli1, and Gli2) were assessed using qRT‐PCR and Western blot. Bioinformatics and qRT‐PCR analyses revealed MYB overexpression in cervical cancer. Signaling pathway prediction indicated MYB enrichment in cytotoxic signaling pathways. Functional experiments demonstrated that MYB overexpression activated the hedgehog signaling pathway, thereby suppressing NK cell cytotoxicity in cervical cancer. Rescue experiments using the hedgehog signaling inhibitor GANT58 attenuated the suppressive effect of MYB overexpression on NK cytotoxicity. In summary, MYB inhibited NK cell cytotoxicity by activating the hedgehog signaling pathway in cervical cancer, suggesting its potential as a novel diagnostic marker and immunotherapeutic target.

## 1 Introduction

Cervical cancer is the second leading cause of cancer‐related deaths in developing countries and one of the most frequently diagnosed malignancies among women worldwide [1]. Prior to cancer development, patients may present with various cervical lesions, including cervical intraepithelial neoplasia (CIN1, CIN2, CIN3) and invasive carcinoma [2]. While human papillomavirus (HPV) infection is the primary risk factor for cervical cancer [3], other contributing factors, such as steroidal sex hormones, oral contraceptive use, and smoking, also promote the transformation of normal cells into malignant ones [4, 5, 6]. Although standard treatments like surgery and radiotherapy have improved survival rates, a subset of patients still experiences poor outcomes. Consequently, developing more effective therapeutic strategies to enhance patient survival has become a critical area of research. Natural killer (NK) cells, a subset of lymphocytes, eliminate cancer cells through antibody‐dependent cell‐mediated cytotoxicity (ADCC). Given their potent antitumor activity, NK cell‐based immunotherapy holds significant promise for cervical cancer treatment [7]. Increased attention has been paid to the molecular mechanisms by which NK cells kill cervical cancer cells. Lirilumab and Avelumab enhance the anti‐HPV+ cervical cancer activity of NK cells via Vav1‐dependent NF‐*κ*B disinhibition [8]. Sato et al. [9] reported that NK cells are activated to suppress cervical cancer progression by downregulating indoleamine‐2,3‐dioxygenase. These findings have demonstrated the role of NK cells in repressing cervical cancer progression, but numerous molecular targets affecting the killing toxicity of NK cells remain underexplored. This knowledge gap has motivated our investigation into actionable targets that modulate NK cell activity in cervical cancer, with the ultimate goal of establishing a conceptual foundation for improved therapeutic strategies.

MYB (c‐MYB), a prototypical transcription factor of the MYB family (which includes MYB, MYBL1, and other members) [10], regulates multiple signaling pathways to control cell survival, growth, and differentiation, making it a key regulator of multiple carcinogeneses [11, 12, 13]. Emerging evidence highlights that MYB drives oncogenic growth in multiple tumors, including cervical cancer. Xie et al. [14] demonstrated that lncRNA SNHG3 establishes oncogenic events like cell proliferation and metastasis by indirectly upregulating the target gene MYB by binding miR‐139‐5p in gastric cancer. Li et al. [15] showed that extracellular vesicle‐encapsulated miR‐424 suppresses tumorigenesis and angiogenesis in ovarian cancer by downregulating MYB. Du et al. [16] showed that miR‐195 inhibits cell malignant phenotypes in cervical cancer by downregulating CCND2 and MYB. Additionally, MYB exerts a modulatory effect on NK cell activity. As illustrated by Lee et al. [17], resveratrol stimulates NK cell activity by activating Akt and mTORC2 signaling pathways to upregulate MYB. However, it is elusive whether MYB is implicated in NK cytotoxicity in cervical cancer. Given this knowledge gap, this study endeavored to delineate the influence of MYB on NK cytotoxicity and its mechanism in cervical cancer.

MYB expression, as well as the impact of MYB on the role and mechanism of NK cytotoxicity in cervical cancer, was investigated. The observations supported the view that MYB was in high‐expression status and activated the hedgehog signaling pathway to suppress the killing of NK cells on cervical cancer cells. Considering the findings, MYB may act as an immunotherapeutic target for NK cell‐based elimination of cervical cancer cells.

## 2 Materials and Methods

### 2.1 Bioinformatics Analysis

The mRNA expression data (normal: 3, tumor: 306) of cervical cancer were downloaded from The Cancer Genome Atlas (TCGA). Differentially expressed mRNAs (DEmRNAs) were identified by differential analysis using edgeR (|logFC| > 2, padj < 0.05). Target genes were subjected to pathway enrichment analysis using GSEA. Correlation of DEGs with genes related to enrichment pathways was analyzed by Pearson coefficient.

### 2.2 Cell Culture

HUCEC (YS1079C), a human normal cervical epithelial cell line, was purchased from Shanghai Yaji Biological. Caski (BFN60700201). Cervical cancer cell lines HeLa (BFN60700111) and SiHa (BFN60700394) were obtained from ATCC (Shanghai, China). Cells were incubated in Dulbecco's modified eagle's medium (DMEM) with 10% fetal bovine serum (FBS), penicillin (100 U/mL), and streptomycin (100 mg/mL) in an incubator at 37°C and 5% CO_2_ [18].

Human NK cells (NK92, BFN608006464, ATCC) were maintained in RPMI‐1640 complete medium with 10% FBS, 2 mM L‐glutamine, 100 U/mL penicillin and streptomycin, and 100 U/mL IL‐2 (PeproTech, USA) in an incubator with a stable temperature (37°C) and a stable CO_2_ level (5%) [19].

### 2.3 Cell Transfection

si‐MYB, pcDNA3.1 vector constructs of oe‐MYB, si‐NC, and oe‐NC were provided by GenePharma (China). GANT58 was purchased from Abcam (UK). Cells (2 × 10^5^) were added to 6‐well plates and were transfected with the constructed si‐MYB/oe‐MYB and si‐NC/oe‐NC by Lipofectamine 2000 (Thermo Fisher Scientific, USA). 48 h later, cells were treated with GANT58 for subsequent experiments [20].

### 2.4 qRT‐PCR

Total RNA isolation from cells was performed with TRIzol reagent (Thermo Fisher Scientific, USA). Reverse transcription was performed using the PrimeScript RT Reagent Kit (Takara Biotechnology, China) to convert 1 μg of total RNA into cDNA. Quantification of mRNA expression was performed on an ABI 7500 Sequence Detection System (Thermo Fisher Scientific, USA) using FastStart Universal SYBR Green Master reagent (Merck, USA). The mRNA expression of the target gene was normalized to GAPDH and computed using the 2^−ΔΔCT^ method [21]. Primer sequences were listed in Table 1.

**TABLE 1:** Primer set for qRT‐PCR.

Gene	Primer sequence (5′ → 3′)
MYB	F: GCCAATTATCTCCCGAATCGA
	R: ACCAACGTTTCGGACCGTA
Gli1	F: GGGATGATCCCACATCCTCAGTC
	R: CTGGAGGAGCCCCCCCAGT
Gli2	F: CTGCCTCCGAGAAGCAAGAAG
	R: GCATGGAATGGTGGCAAGAG
PTCH1	F: TGACTCCCAAGCAAAT
	R: ATCCTGATGAACCACCTC
GAPDH	F: CCATGGAGAAGGCTGGG
	R: CAAAGTTG TCATGGATGACC

### 2.5 Western Blot

Collected cells were subject to phosphate buffered saline (PBS) washes, and total proteins were isolated per the instructions of the extraction kit (R&D Systems, China). The protein concentration was determined using the BCA protein assay kit (Thermo Fisher Scientific, USA). Following SDS‐PAGE, total proteins were transferred to PVDF membranes that were blocked with 5% skim milk powder in Tris‐buffered saline with Tween 20 for 2 h. Rabbit anti‐human Gli1 (1:1000, ab134906, Abcam, UK), Gli2 (1:1000, ab277800, Abcam, UK), PTCH1 (1:1000, ab53715, Abcam, UK), GAPDH (1:1000, ab181602, Abcam, UK), Cleaved Caspase‐3 (1:1000, 9661, CST, Danvers, USA), BCL‐2 (1:1000, ab182858, Abcam, UK), BAX (1:1000, ab32503, Abcam, UK), HPV16 E7 (1:1000, sc‐65,711, Santa Cruz, USA), and HPV18 E7 (sc‐365,035, Santa Cruz, USA) primary antibodies were added. After storage overnight at 4°C, horseradish peroxidase‐labeled goat anti‐rabbit IgG (1:2000, ab6721, Abcam, UK) and goat anti‐mouse IgG (1:2000, ab205719, Abcam, UK) secondary antibodies were added and incubated at room temperature for 1 h. After membrane rinses, immunoreactivity was assessed using ECL solution (Thermo Fisher Scientific, USA). Imaging analysis was performed using the ChemiDoc System (Bio‐Rad, USA).

### 2.6 CCK‐8 Assay

CCK‐8 (Solarbio, China) was recommended to test cell viability. 100 μL of cell suspension was plated into a 96‐well plate. Following 0, 24, 48, and 96 h of culture at 37°C, cells were cultured for 2 h at 37°C protected from light with the addition of 10 μL CCK‐8 reagent. The absorbance at 450 nm was tested with a full‐wavelength multifunctional enzyme labeling instrument (TECAN, Switzerland).

### 2.7 Cytotoxicity Assay

NK cell impact on killing cervical cancer cells was assayed using the CytoTox 96 non‐radioactive cytotoxicity assay (Promega, USA). HeLa and SiHa cells were plated into 96‐well microplates (5 × 10^3^ cells/well). At a 10:1 ratio of effector to target cells (E:T), IL‐2‐induced NK cells (5 × 10^4^) were cocultured with HeLa and SiHa cells, severally, followed by 4 h of culture at 37°C. Cell mixture‐sourced supernatants were produced by centrifugation. NK cytotoxicity against cervical cancer cells was computed by the standard formula: Cytotoxicity = (Experimental‐Effector spontaneous‐Target Spontaneous)/(Target maximum‐Target spontaneous) × 100% [19].

### 2.8 ELISA

IFN‐*γ* and TNF‐*α* levels were assessed with Human IFN‐*γ* ELISA Kit (Abcam, USA) and Human TNF‐*α* ELISA Kit (Abcam, USA) [19]. All experimental procedures were strictly performed according to the manufacturer's instructions. After terminating the reaction, the absorbance was measured at 450 nm using a microplate reader.

### 2.9 Immunofluorescent Analysis

NK cells were fixed in 4% formalin at 4°C for 8 h, and paraffin‐embedded blocks were created and sliced into 3 μm sections, which were mounted on slides. Slides were transparent in 0.2%–0.5% Triton X‐100 and blocked for 1 h at room temperature in 5% normal donkey serum. Slides were incubated overnight with antiperforin and anti‐granzyme B antibody (Abcam, UK), followed by DAPI and fluorescein‐conjugated goat anti‐rabbit IgG (Abcam, UK). Slides were fixed with fluorescent mounting media and imaged by an optical microscope.

### 2.10 Cell Apoptosis Analysis

Apoptotic cells were assayed by annexin V‐FITC and PI staining with the FITC‐Annexin V Apoptosis Detection Kit with PI (BioLegend, USA). The cells were resuspended and prepared as single‐cell suspensions at a density of 1 × 10^6^ cells/mL. A 100 μL aliquot of cell suspension was sequentially stained with Annexin V‐FITC and PI staining solutions, followed by 15 min of incubation at room temperature in the dark. The reaction was terminated by adding Binding Buffer. The experimental setup included a negative unstained control, a single‐stained control, and a positive apoptosis control group (all operations were performed at 4°C in the dark, with detection completed within 2 h after staining). Detection was carried out using the Accuri C6 flow cytometer (BD, USA) [22].

### 2.11 Statistical Analysis

Data were presented as mean ± SD and processed on SPSS software (version 18.0). Differences between groups were compared by Student's *t*‐test or analysis of variance, and Pearson's coefficient was employed for correlation analysis. *p* < 0.05 represented statistically significant.

## 3 Results

### 3.1 MYB is in High‐Expression Status in Cervical Cancer

**FIGURE 1:**
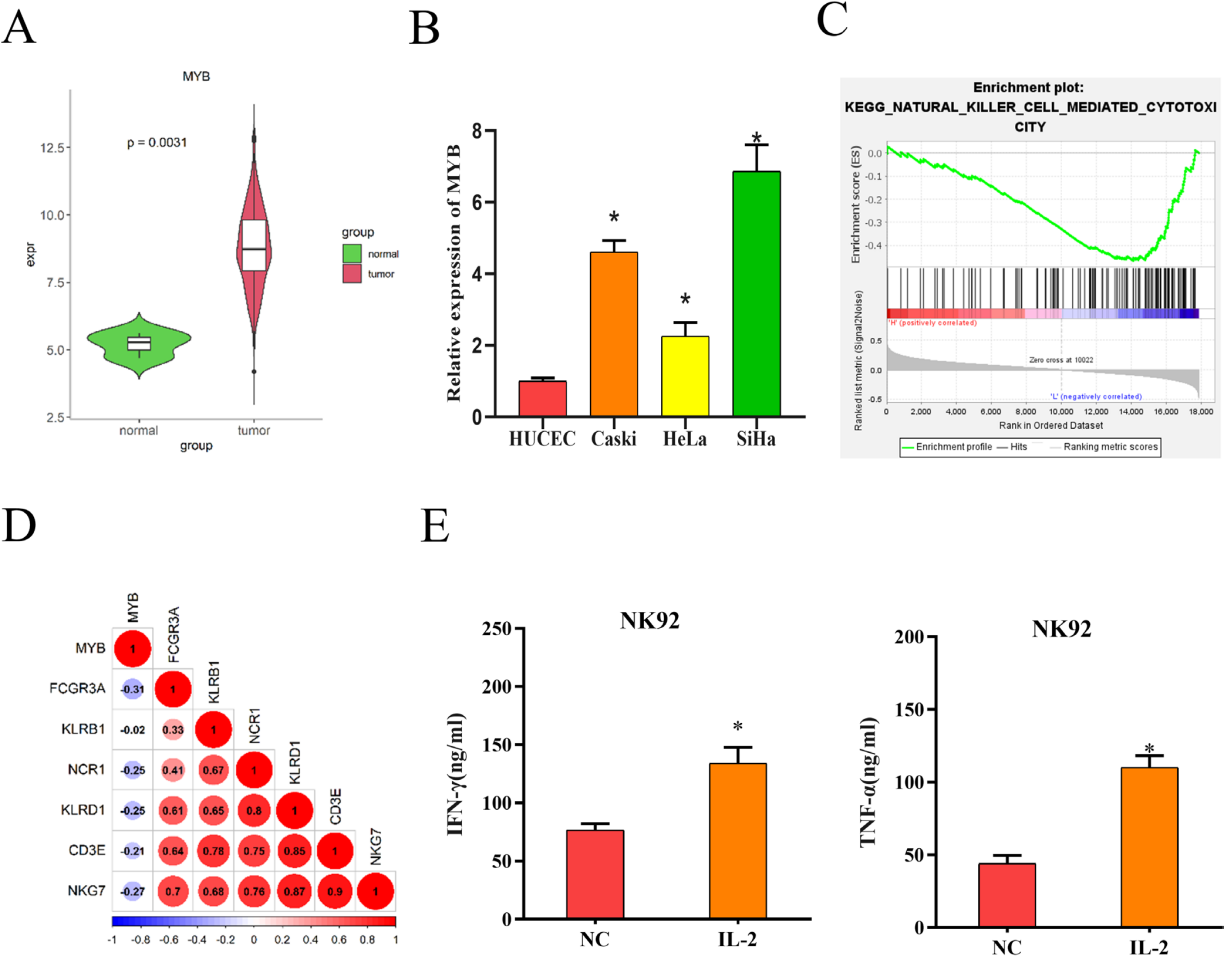
MYB is in high‐expression status in cervical cancer. (A) Prediction of MYB expression in cervical cancer tissues and normal tissues through TCGA database. (B) QRT‐PCR detection of MYB expression in cervical cancer cell lines and a human normal cervical epithelial cell line. (C) GSEA pathway analysis of MYB. (D) Pearson correlation analysis of MYB with NK cytotoxicity‐related genes. (E) ELISA of IFN‐*γ* and TNF‐*α* expression levels in IL‐2 activated NK cells. *p < 0.05.

Differential analysis of data from TCGA yielded 2373 DEmRNAs (1017 upregulated and 1356 downregulated), inclusive of MYB. MYB is a proto‐oncogene and is upregulated in cancers like cervical cancer, manipulating cancer progression [23, 24]. *T*‐test results revealed that MYB level was substantially higher in cervical cancer tissues than in normal tissues (Figure 1A). MYB levels in HUCEC, Caski, HeLa, and SiHa cells were assayed by qRT‐PCR, which revealed a marked high expression of MYB in cervical cancer cells (Figure 1B). Thus, MYB was selected as the gene of interest. SiHa cells with the highest MYB level and HeLa cells with the lowest level were utilized for subsequent experiments. The enrichment of MYB was exhibited in the NK cell‐mediated cytotoxicity signaling pathway as analyzed by GSEA (Figure 1C). Pearson correlation analysis revealed significant negative correlations between MYB and NK cytotoxicity‐related genes KLRB1, CD3E, NKG7, NCR1, KLRD1, and FCGR3A (Figure 1D). On these grounds, we speculated that MYB was implicated in NK cytotoxicity in cervical cancer. NK cells were stimulated with IL‐2 (20 ng/mL) for 24 h, and then secretion levels of cytotoxic factors IFN‐*γ* and TNF‐*α* were assessed by ELISA. IFN‐*γ* and TNF‐*α* levels were elevated in NK cells, indicating an activation of NK cells, which could be utilized for subsequent experiments (Figure 1E). These data provided evidence that MYB was in high‐expression status and may affect NK cytotoxicity in cervical cancer.

### 3.2 MYB High Expression Represses NK Cell Impact on Killing Cervical Cancer Cells

**FIGURE 2:**
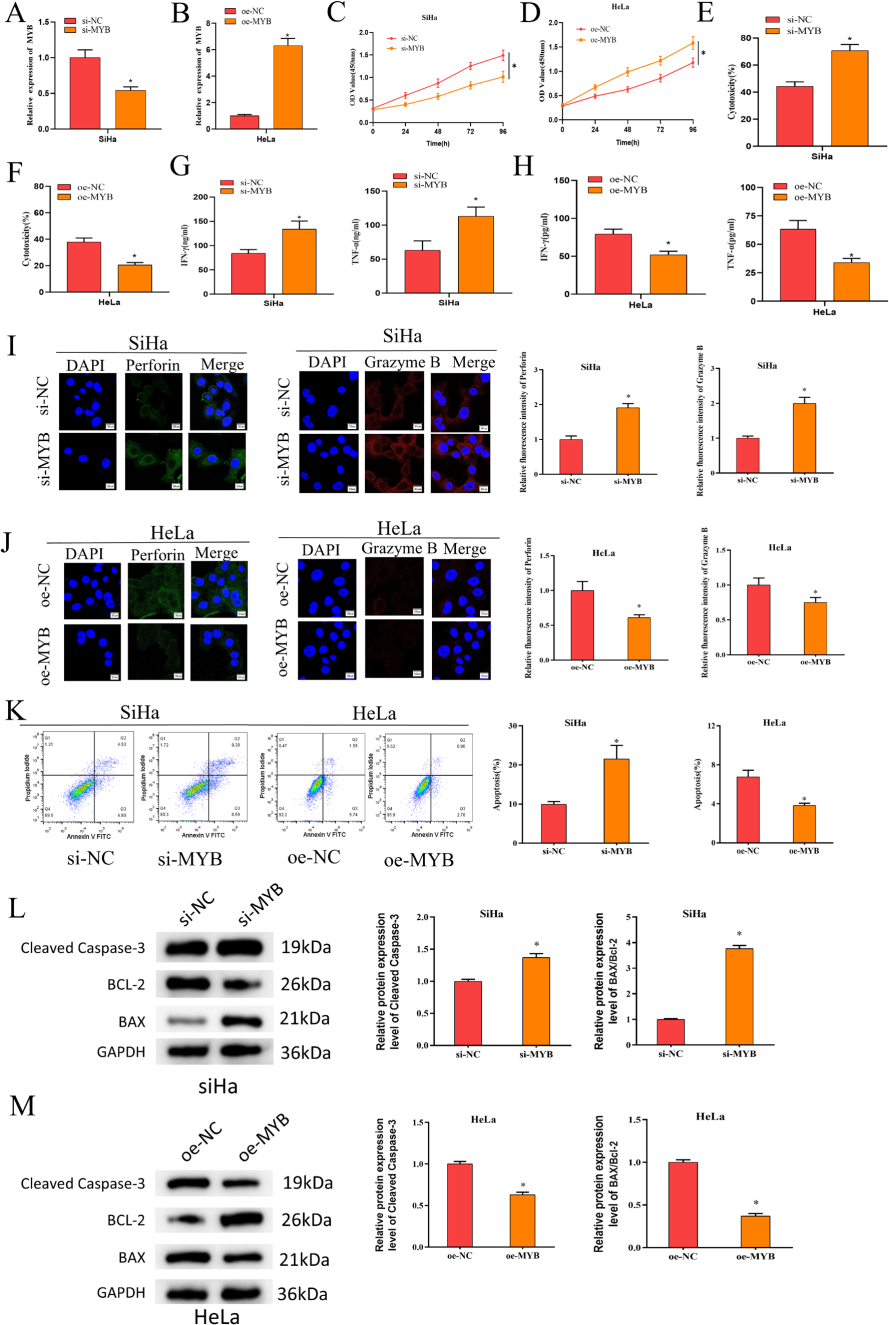
MYB high expression represses killing effect of NK cells on cervical cancer cells. (A, B) Detection of transfection efficiency by qRT‐PCR. (C, D) Detection of cell viability after transfection by CCK‐8. (E, F) Detection of the killing effect of NK cells on cervical cancer cells after transfection by cytotoxicity assay. (G, H) Detection of levels of IFN‐*γ* and TNF‐*α* released from NK cells after transfection by ELISA. (I, J) Detection of levels of perforin and granzyme B released from NK cells after transfection by immunofluorescence. (K) Detection of apoptosis rate of cervical cancer cells after transfection by flow cytometry. (L, M) Western blot detection of the expression of apoptosis‐related and anti‐apoptotic proteins. *p < 0.05.

To investigate the influence of MYB on NK cytotoxicity in cervical cancer, si‐NC/si‐MYB; oe‐NC/oe‐MYB cell groupings were generated based on SiHa and HeLa. Transfection efficiency as tested by qRT‐PCR unveiled that compared with the control, knockdown of MYB suppressed MYB expression in SiHa cells, but upregulation of MYB upregulated MYB expression in HeLa cells (Figure 2A,B), suggesting favorable transfection efficiency. CCK8 results illustrated that knockdown of MYB notably suppressed SiHa cell viability, but upregulation of MYB significantly increased HeLa cell viability compared to the control group (Figure 2C,D). NK cell impact on killing cervical cancer cells was detected by cytotoxicity assay using successfully transfected cervical cancer cells cocultured with activated NK cells. Knockdown of MYB significantly increased NK cytotoxicity, but upregulation of MYB significantly decreased NK cytotoxicity compared to the control (Figure 2E,F). ELISA results reported that compared with the control, levels of cytotoxic factors IFN‐*γ* and TNF‐*α* released from NK cells were significantly elevated in the SiHa group with knockdown of MYB, but NK cell‐released IFN‐*γ* and TNF‐*α* levels were significantly decreased in the HeLa group with upregulation of MYB (Figure 2G,H). Levels of perforin and granzyme B released from NK cells showed a significant increase in the SiHa group with knockdown of MYB and a significant decrease in the HeLa group with upregulation of MYB, as detected by immunofluorescence (Figure 2I,J). By flow cytometry, the apoptosis rate of SiHa cells with knockdown of MYB was found to be significantly increased, but that of HeLa cells with upregulation of MYB was found to be significantly decreased compared to the control (Figure 2K). Subsequently, the expression levels of apoptosis/anti‐apoptosis‐related proteins were detected by western blot. Compared with the control group, MYB‐knockdown SiHa cells exhibited significantly increased levels of Cleaved Caspase‐3 and Bax/Bcl‐2, whereas MYB‐overexpressing HeLa cells showed significantly decreased levels of Cleaved Caspase‐3 and Bax/Bcl‐2 (Figure 2L,M). Together, these findings revealed that high MYB expression inhibited the NK cell impact on killing cervical cancer cells. Furthermore, to determine whether si‐MYB or oe‐MYB affects the expression of the HPV E7 oncoprotein, we examined HPV E7 levels in MYB‐knockdown or MYB‐overexpressing SiHa and HeLa cells (cervical cancer cell lines harboring integrated HPV16 and HPV18 DNA, respectively). The results showed that MYB knockdown significantly reduced HPV16 E7 oncoprotein levels, whereas MYB overexpression significantly increased HPV18 E7 oncoprotein levels (Figure S1A,B).

### 3.3 MYB High Expression Activates the Hedgehog Signaling Pathway

**FIGURE 3:**
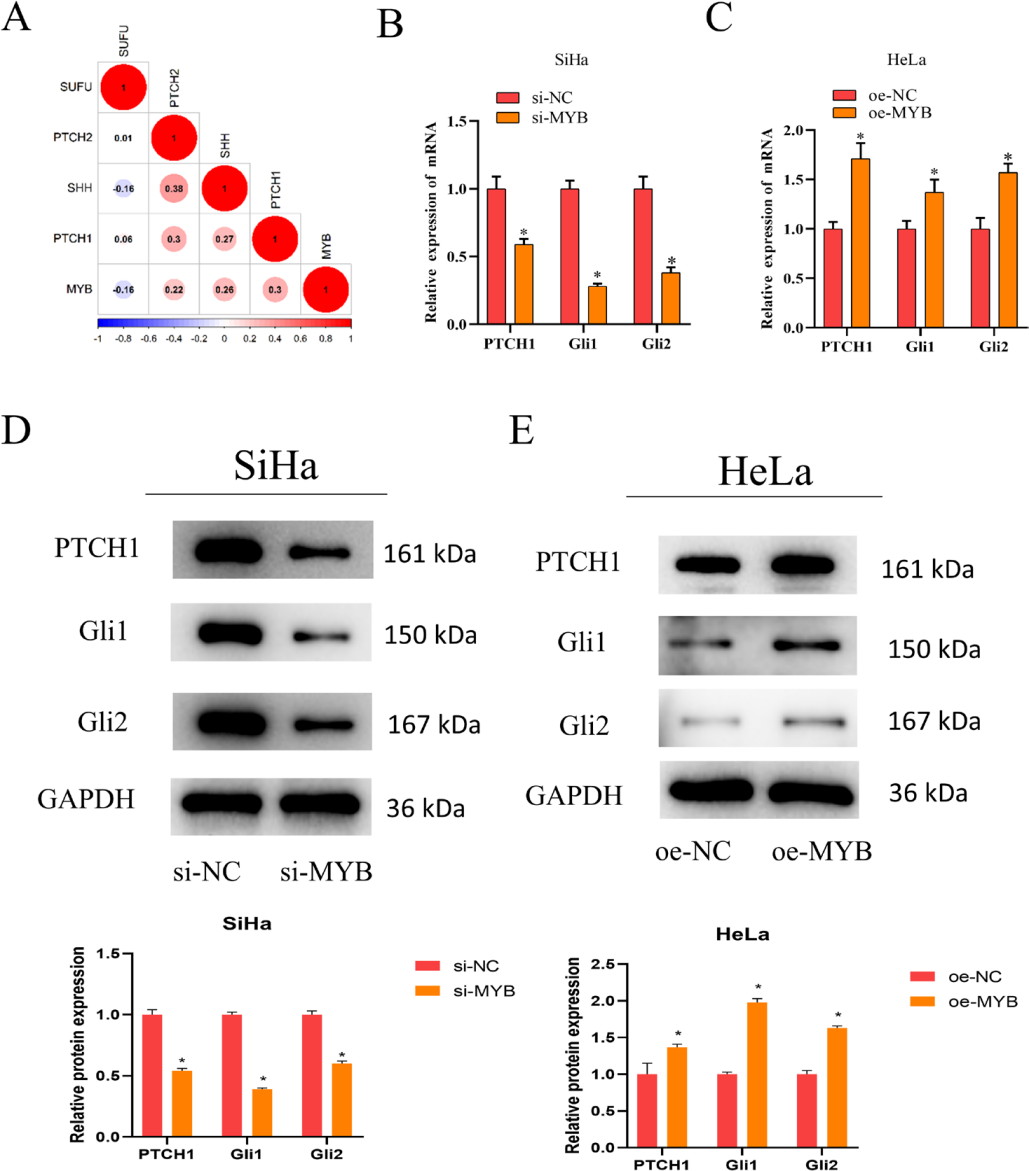
MYB high expression activates hedgehog signaling pathway. (A) Pearson correlation analysis of MYB with hedgehog signaling pathway‐related genes. (B, C) QRT‐PCR analysis of mRNA expression of hedgehog signaling pathway‐related factors PTCH1, Gli1, and Gli2. (D, E) Western blot analysis of protein expression of hedgehog signaling pathway‐related factors PTCH1, Gli1, and Gli2. *p < 0.05.

MYB activates the hedgehog signaling pathway and thus manipulates cancer progression [25]. To delineate the impact of MYB on the hedgehog signaling pathway in cervical cancer cells, Pearson correlation analysis was conducted. MYB expression was positively correlated with PTCH1, PTCH2, and SHH, the marker genes of the hedgehog signaling pathway (Figure 3A). The mRNA levels of hedgehog signaling pathway‐related factors (PTCH1, Gli1, and Gli2) were assessed using qRT‐PCR. Knockdown of MYB inhibited mRNA expression of PTCH1, Gli1, and Gli2 in SiHa cells, whereas upregulation of MYB exerted an inverse effect in HeLa cells (Figure 3B,C). Protein levels of PTCH1, Gli1, and Gli2 were assayed via western blot. Knockdown of MYB significantly inhibited protein expression of these factors in SiHa cells, but upregulation of MYB showed the opposite effect in HeLa cells (Figure 3D,E). These data supported that MYB high expression activated the hedgehog signaling pathway.

### 3.4 MYB Represses NK Cell Impact on Killing Cervical Cancer Cells Through the Hedgehog Signaling Pathway

**FIGURE 4:**
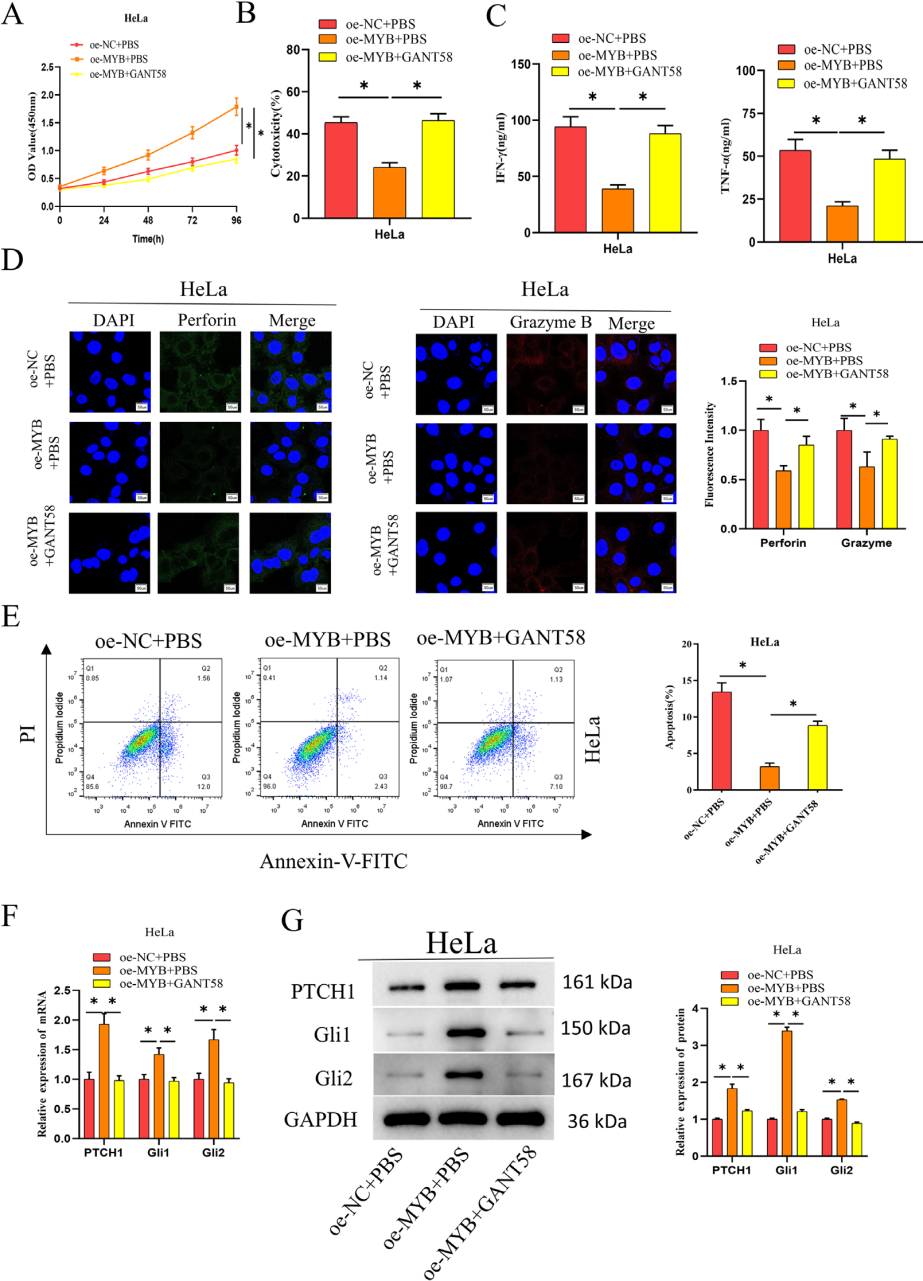
MYB represses the killing effect of NK cells on cervical cancer cells by activating the hedgehog signaling pathway. (A) Viability of cervical cancer cells after transfection as assayed via CCK‐8. (B) The killing effect of NK cells on HeLa cells after transfection as tested by cytotoxicity assay. (C) Expression of cytotoxic factors IFN‐*γ* and TNF‐*α* released by NK cells after transfection as assessed through ELISA. (D) Expression of toxic molecules perforin and granzyme B released by NK cells after transfection as measured via immunofluorescence. (E) Apoptosis rate of HeLa cells after transfection as detected through flow cytometry. (F, G) Expression of hedgehog signaling pathway‐related factors PTCH1, Gli1, and Gli2 after transfection as assayed via qRT‐PCR and western blot. *p < 0.05.

Activation of the hedgehog signaling pathway suppresses intra‐tumor NK cytoactive [26]. To elucidate whether MYB represses the killing effect of NK cells by activating the hedgehog signaling pathway, oe‐NC + PBS, oe‐MYB + PBS, and oe‐MYB + GANT58 (hedgehog signaling pathway inhibitor) groups were constructed in HeLa cells. The results of CCK8 reported that the upregulation of MYB significantly facilitated HeLa cell viability compared to the oe‐NC + PBS group, while the addition of GANT58 weakened the stimulation to HeLa cell viability by high MYB expression (Figure 4A). By using successfully transfected cervical cancer cells cocultured with activated NK cells, the cytotoxicity assay detected the NK cell impact on killing cervical cancer cells. The toxicity of NK cells with high MYB expression was significantly reduced compared to the oe‐NC + PBS group, while this effect was attenuated by adding GANT58 (Figure 4B). The expression of cytotoxic factors TNF‐*α* and IFN‐*γ* released from NK cells was measured by ELISA. A significant decrease in TNF‐*α* and IFN‐*γ* levels was observed in NK cells with high MYB expression but not in the oe‐NC + PBS group, while the addition of GANT58 weakened the repressive effect (Figure 4C). Levels of perforin and granzyme B released from NK cells were assayed via immunofluorescence. The content of perforin and granzyme B in NK cells with high MYB expression was significantly reduced compared to the oe‐NC + PBS group, and this inhibitory effect was attenuated by the addition of GANT58 (Figure 4D). The apoptosis level of tumor cells after coculture with activated NK cells was tested through flow cytometry. HeLa cells in the coculture system had less apoptosis property in the case of high MYB expression than the oe‐NC + PBS group, whereas the suppressive effect was reduced by adding GANT58 (Figure 4E). Levels of hedgehog signaling pathway‐related factors PTCH1, Gli1, and Gli2 were tested via qRT‐PCR and western blot. MYB high expression markedly increased mRNA and protein expression of PTCH1, Gli1, and Gli2 in HeLa cells compared to the oe‐NC + PBS group, whereas the addition of GANT58 attenuated the stimulatory effect (Figure 4F,G). The above data suggested that MYB repressed the NK cell impact on killing cervical cancer cells through activation of the hedgehog signaling pathway.

## 4 Discussion

Cervical cancer ranks as the second most frequent gynecologic malignancy globally [27]. Immunotherapy, considering the immune microenvironment, opens up a new arena for the management of cervical cancer. NK cells are an indispensable component of the tumor microenvironment and play a prominent role in anti‐tumor immunity. For instance, in vitro experiments on cervical cancer displayed that the use of vorinostat (a histone deacetylase inhibitor) facilitates an increase in MICA expression, thereby enhancing NK cell toxicity against cervical cancer cells [28]. Zhu et al. [19] manifested that miR‐20a inhibits NK cell impact on killing cervical cancer cells through downregulation of RUNX1. Data in this investigation supported that MYB was in high‐expression status and was able to repress the NK cell impact on killing cervical cancer cells.

MYB is a proto‐oncogene, belonging to the MYB transcription factor family [29]. It was initially thought to exert a crucial effect in the modulation of hematopoiesis and tumorigenesis [30]. High MYB expression is associated with the malignant progression of pancreatic cancer [31], glioma [32], and cervical cancer [16]. Sun et al. [29] reported that miR‐150 represses melanoma tumor growth by downregulating MYB. Xie et al. [14] found that lncRNA SNHG3 indirectly upregulates MYB by targeting miR‐139‐5p in gastric cancer and thus stimulates proliferation and metastasis. However, a closer examination of the uncharacterized function and mechanism of MYB in cervical cancer is warranted. In this study, we generated a result that high MYB expression suppressed the NK cell impact on killing cervical cancer cells through cell experiments. This finding bolstered our understanding of cervical cancer pathogenesis and suggested that targeting MYB may be a possible target for immunotherapy on the basis of NK cells.

To investigate the specific mechanism by which MYB high expression repressed NK cell toxicity, bioinformatics analysis was done, and it revealed a strong positive correlation of MYB with marker genes of the hedgehog signaling pathway. Cell experiments were completed, and their results could be concluded that MYB high expression activated the hedgehog signaling pathway. Hedgehog signaling molecules are signaling cell‐secreted local protein ligands whose signaling pathways control cell survival, proliferation, and differentiation [33]. Aberrant hedgehog signaling pathway transmission is implicated in the development of cervical cancer, and the inhibition of the hedgehog signaling pathway hinders cancer progression. Tetramethylpyrazine suppresses the proliferation, invasion, and migration of C33A cells, a cervical cancer cell line, by blocking the hedgehog signaling pathway [34]. Sharma et al. [35] manifested that GANT58 suppresses cervical cancer epithelial‐mesenchymal transition by blocking the hedgehog signaling pathway. Additionally, activation of the hedgehog signaling pathway has been proposed to suppress the killing effect of NK cells. As revealed by Cascio et al. [26], cancer‐associated mesenchymal stem cells inhibit NK cytotoxicity in ovarian cancer by activating the hedgehog signaling pathway, thus improving resistance to immunotherapy. To complete our findings, rescue experiments were performed. MYB was observed to restrain NK cell impact on killing cervical cancer cells through activation of the hedgehog signaling pathway. Previously, no study reported the specific mechanism behind MYB affecting NK cell killing in cervical cancer, and thus, this result contributed to the understanding of the molecular mechanism and provided avenues for identifying therapeutic targets in cervical cancer.

In conclusion, these findings evidenced, for the first time, that MYB repressed NK cytotoxicity in cervical cancer, and such repression was achieved by activating the hedgehog signaling pathway. Thus, MYB was nominated as a possible immunotherapeutic target for cervical cancer, and suppression of MYB may be an alternative way to activate NK cell‐mediated elimination of tumor cells. However, this study also has certain limitations, such as the constraints of the cell models. The research was based on HPV‐positive cervical cancer cells (e.g., SiHa, HeLa) but did not include HPV‐negative cells (e.g., C33A) or other cervical cancer subtypes, which may affect the generalizability of the conclusions. In the future, we will incorporate HPV‐negative control cell lines as well as other cervical cancer subtype cell lines to more comprehensively analyze the similarities and differences between HPV‐dependent and HPV‐independent mechanisms. When investigating the impact of MYB on NK cell killing activity, we only explored the effects of MYB overexpression or knockdown on NK92 cytotoxicity. Future studies could also examine MYB expression in NK cells from cervical cancer patients to further validate our findings. Secondly, although this study demonstrates that MYB activates the Hedgehog pathway, the specific regulatory nodes remain unclear. Future research will further explore whether MYB directly binds to the promoter regions of key genes in the Hedgehog pathway. These are all important directions for our future studies. We plan to conduct in vivo animal experiments and collect more clinical samples for more systematic analysis and validation. In conclusion, our findings provide evidence in favor of MYB being a possible immunotherapeutic target for cervical cancer. Targeting the MYB‐Hh pathway may also provide new insights for enhancing immune cell function.

## Conflicts of Interest

The authors declare no conflicts of interest.

## Supporting information


**Figure S1.** MYB affects the expression of HPV E7 oncoprotein. (A) Western blot analysis of HPV16 E7 expression in MYB‐knockdown SiHa cells. (B) Western blot analysis of HPV18 E7 expression in MYB‐overexpressing HeLa cells; *p < 0.05.

## Data Availability

The data that support the findings of this study are available from the corresponding author upon reasonable request.
