# Supplementary figures and images for: MYB Activates the Hedgehog Signaling Pathway to Repress Natural Killer Cytotoxicity in Cervical Cancer
Source: Kaohsiung J Med Sci. 2025 Sep 1;41(12):e70084. doi: 10.1002/kjm2.70084 (PMC12694565; doi:10.1002/kjm2.70084)

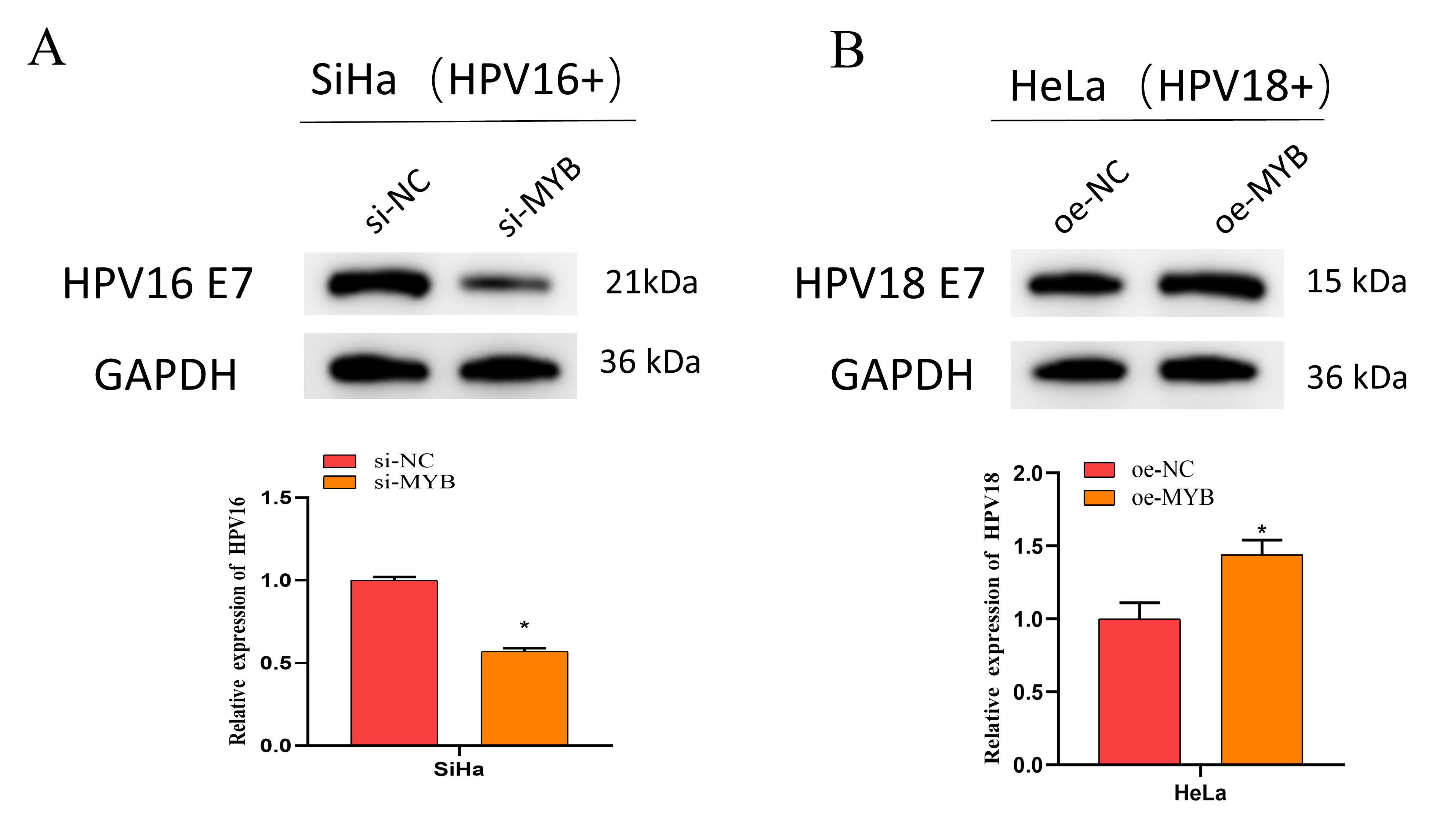

Supplement: Supplementary file 1 — Figure S1. MYB affects the expression of HPV E7 oncoprotein. (A) Western blot analysis of HPV16 E7 expression in MYB‐knockdown SiHa cells. (B) Western blot analysis of HPV18 E7 expression in MYB‐overexpressing HeLa cells; *p < 0.05. [file KJM2-41-e70084-s001.tif]
